# Cystic adrenal lesions: A report of five cases

**DOI:** 10.1002/cnr2.1314

**Published:** 2020-12-09

**Authors:** Divya Goel, Loreno Enny, Chanchal Rana, Pooja Ramakant, Kulranjan Singh, Suresh Babu, Anand Mishra

**Affiliations:** ^1^ Department of Pathology King George's Medical University Lucknow Uttar Pradesh India; ^2^ Department of Endocrine surgery King George's Medical University Lucknow Uttar Pradesh India

**Keywords:** adrenal cancer, adrenal cyst, adrenalectomy, diagnosis

## Abstract

**Background:**

Cystic adrenal lesions are rare and uncommon manifestation with few cases reported so far. Different types of adrenal cysts have been described with heterogeneous etiology and overlapping clinical findings, ranging from benign to malignant cystic neoplasm. They are usually asymptomatic or may rarely present with abdominal pain or fullness. Optimum management of adrenal cysts still remain controversial, owing to its low incidence. In this study, we report our institutional experience on diagnosis and management of different histological types of cystic adrenal lesions.

**Cases:**

During 4 years period, 55 patients underwent adrenalectomy with five cases presenting as adrenal cysts. All the five patients were biochemically nonfunctional and underwent adrenalectomy (laparoscopic anterior n = 2, retroperitoneoscopic approach n = 1, and open anterior transperitoneal approach n = 2). The primary indications for surgery were larger size and/or suspicion of malignancy. Histological evaluation revealed two epithelial cysts, one endothelial cyst, one pseudocyst, and a very rare case of adrenocortical carcinoma arising in a pseudocyst.

**Conclusion:**

Cystic adrenal lesions are rare with varied etiologic and clinical presentation that may sometimes lead to diagnostic and management dilemma. These cases must undergo biochemical and radiological evaluation to rule out underlying malignancy followed by referral for surgical intervention.

## 1 INTRODUCTION

Cystic adrenal lesions are uncommon with first case reported as early as 1670.^1^ They often present with nonspecific clinical and radiological features, hence remain under recognized. Benign adrenal cysts are very rare lesions that are incidentally discovered on imaging examinations, with a prevalence of ~1% among adrenal lesions.^2^ Traditionally, adrenal gland cysts have been classified as pseudocysts, endothelial cysts, epithelial cysts, and parasitic cysts.^2^ However, small series of cystic neoplasm have also been reported in past. These cystic lesions have detrimental clinical consequences when associated with malignant neoplasm but mimicking benign lesions. Only few cases series on cystic adrenal lesions have been published so far with none from India.^3^, ^4^, ^5^, ^6^ Management algorithms for adrenal cysts also vary and are controversial because of the overall rarity of such lesions.^6^

The goals of the current study were to review the characteristics of adrenal cysts from a single institution that were surgically removed along with detailed discussion on clinical, radiological, and management aspects.

## 2 CASES

This is a retrospective descriptive study including all the patients who visited the Department of Endocrine Surgery (between June 2016 and December 2020) in King George's Medical University, Lucknow in Uttar Pradesh, India with an adrenal mass and were referred to Department of Pathology for histopathological evaluation. Hematoxylin and eosin‐stained sections were reviewed by two pathologists for verification of diagnosis. Macroscopic pathologic features were obtained through review of the pathology reports, macroscopic photographs, and surgical reports. Clinical data as well as pathological details were retrieved from the pathology and clinical files. The adrenal masses which were cystic in nature were identified and studied in detail as well as described in result section of this article. Fifty‐five cases of adrenal mass were evaluated histopathologically for various adrenal pathologies in the Department of Pathology, King George's Medical University between June 2016 and March 2020. Mean age of the cohort was 36 years (age range 1.5‐63 years) with female predominance (M:F = 1:1.7). There were five unusual cases of cystic adrenal lesions including two cases of epithelial adrenal cyst as well as one case each of endothelial cyst, pseudocyst, and a very rare case of adrenocortical carcinoma arising from a pseudocyst. These cases are described below.

Case 1 A 48‐year‐old male who was under evaluation for abdominal fullness and dyspepsia from medical gastroenterology where he was diagnosed to have left adrenal lesion was referred to our department for further evaluation. He had no history suggestive of a functional adrenal mass. The patient had no comorbidities or significant past history. On clinical examination, abdomen was soft with no evidence of any organomegaly. His upper GI endoscopy revealed normal study. Serum morning cortisol (5.60 μg/dl, reference range is 5‐23 μg/dl) and 24 hours urinary metanephrine (95.40 μg, normal level <350 μg/24 hours) and nor metanephrine (157.44 μg, normal level <600 μg/24 hours) levels were also within normal limits.

**FIGURE 1:**
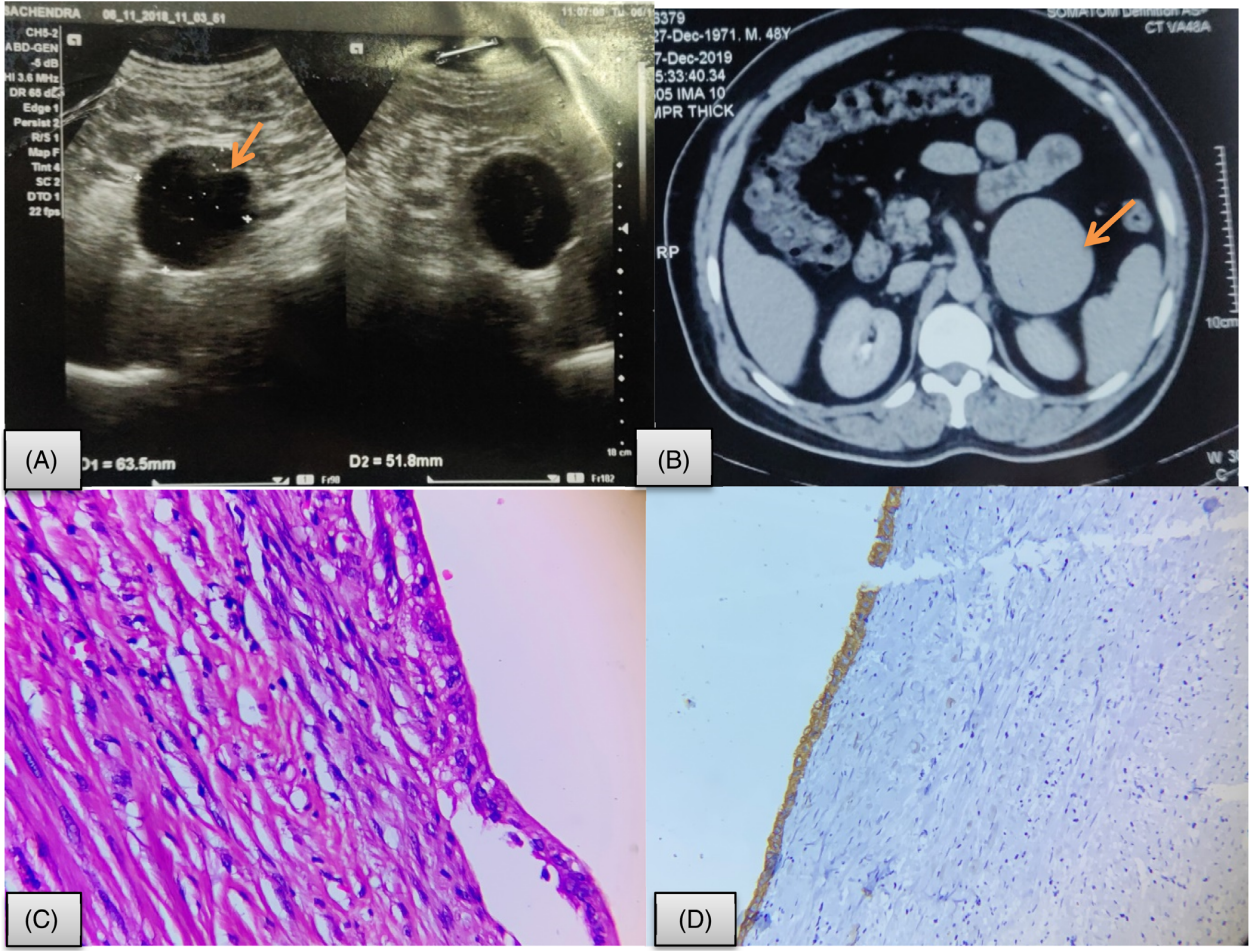
Epithelial adrenal cyst. A, USG abdomen showing (arrow) a well‐defined hypo‐echoic lesion in left suprarenal region measuring 64 × 52 mm (? adrenal origin); B, CECT showing a well defined, round to oval, hypo‐dense lesion with hyper dense thin wall measuring 6.3 × 6.4 × 6.6 cm in the retroperitoneum closely abutting pancreas (arrow); C, microphotograph displaying a cyst with fibro collage nous cyst wall lined by single layer of cuboidal epithelial cells (Hematoxylin and eosin; 40×); D, Immunohistochemistry shows CK positivity in lining epithelial cells (Hematoxylin and eosin; 400×)

Computerized tomography (CT) scan of abdomen revealed well defined, round to oval, hypo dense lesion with hyper dense thin wall measuring 6.3 × 6.4 × 6.6 cm in the retroperitoneum closely abutting pancreas. There was subtle calcification in inferior aspect however no enhancing solid component was identified. Although the patient had no significant symptoms, since the size of the lesion was >6 cm, surgery was planned and he was managed by laparoscopic adrenalectomy via anterior approach. Preoperatively, there was a well‐circumscribed cystic mass lesion, filled with serous fluid, at left suprarenal region. Grossly, the outer surface was smooth, congested, and covered with fibro fatty tissue. Histopathological evaluation revealed a cystic cavity variable wall thickness (2‐3 mm) composed of fibrocollagenous wall which was lined by flattened epithelial cells along with remnants of adrenocortical tissue at the periphery (Figure 1). Hence, the final diagnosis of epithelial adrenal cyst was made. There was no evidence of parasite or malignancy. The postoperative period was uneventful and the patient is in follow for last 6 months.

Case 2 A similar case of 38‐year‐old female patient presented with chief complain of pain in hypochondria, which radiated to back, for 2 years. Serum morning cortisol level was 5.84 μg/dl. Twenty‐four hours urinary metanephrine and normetanephrine levels were 25.90 and 45.93 μg, respectively. CT scan revealed a large well‐defined round to oval non enhancing thin‐walled cystic lesion in suprarenal region on right side leading to significant mass effect to adjacent viscera measuring 66 × 86 × 80 mm. This patient was also managed by laparoscopic adrenalectomy via anterior approach and grossly specimen was received as cystic structure with smooth outer surface weighing 19 g. Final diagnosis was epithelial adrenal cyst.

Case 3 A 35‐year‐old female who was under investigation for hepatic cystic lesion was referred to the Department of Endocrine Surgery for further evaluation. She had initially presented with complaints of pain in right upper abdomen for 5 months which was insidious in onset, dull aching, non‐radiating and was relieved only on medication. There was no abdominal distension, vomiting, altered bowel, or bladder habits. Serum cortisol (11 μg /dl) and 24 hours urinary metanephrine (50.67 μg) and nor metanephrine (250.56 μg) levels were within normal limits. Biochemical investigations also showed normal liver and kidney function tests as well as negative ELISA for Echinococcus. Ultrasonography of the abdomen, which was performed before the referral revealed an irregular cystic shadow, measuring 35 × 33 mm in the posterior segment of right lobe of liver with foci of calcification, suggestive of right hepatic hydatid cyst. CECT of the abdomen revealed a multiloculated cystic lesion measuring 31 × 32 × 36 mm with multiple wall and septal calcification in right adrenal region. Right adrenal gland was not visualized separately from the lesion. The image was suggestive of an adrenal cystic lesion.

**FIGURE 2:**
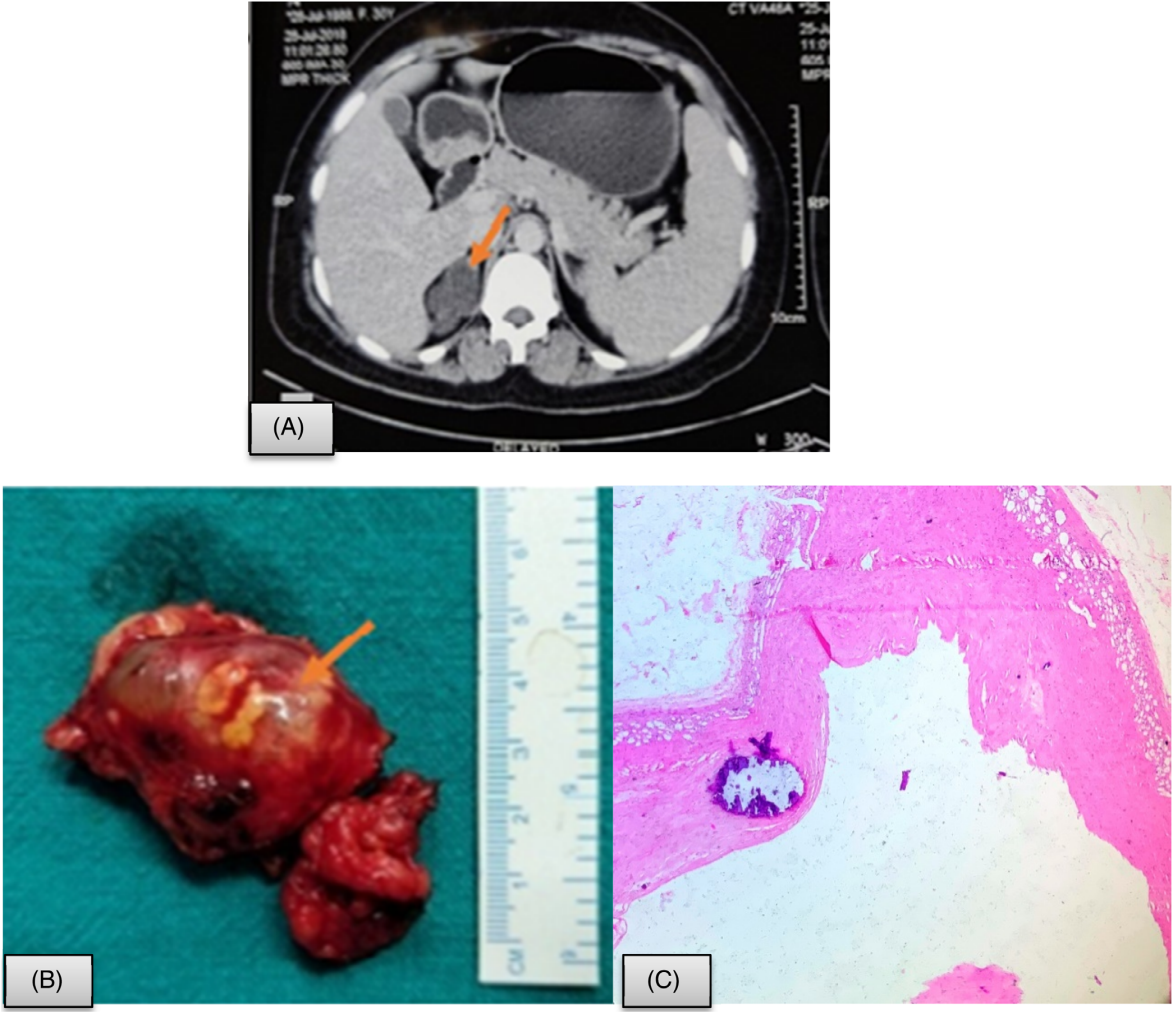
Adrenal pseudo cyst. A, CECT showing a multiloculated cystic lesion measuring 31 × 32 × 36 mm with multiple wall and septal calcification in right adrenal region (arrow); B, Resected specimen showing multiloculated lesion filled with fluid; C, Histopathology shows fibrocollagenous cyst wall with focal calcification and no lining. Remnant of adrenal tissue is seen at the outer aspect (Hematoxylin and eosin; 400×)

In view of patient's symptoms, surgical management was planned and retroperitoneoscopic adrenalectomy was performed. Intraoperatively, the lesion appeared multiloculated containing straw‐colored fluid. It measured 4 × 3 cm in size, with all borders free. Both intraoperative and postoperative periods were uneventful and the patient was discharged on postoperative day 2. The postoperative histopathology showed a cystic lesion with fibrocollagenous wall and no evident lining. Remnant of adrenal tissue is seen at the outer aspect. Histology was consistent with adrenal pseudocyst (Figure 2).

Case 4 A 29‐year‐old normotensive female presented with dull aching pain in right hypochondrial region, radiating to back. She had similar episodes 7 and 3 years back for which she underwent image‐guided aspiration. There was no history of fever, weight loss, visual disturbance, or palpitation. Serum morning cortisol, 24 hours urinary metanephrine and 24 hours urinary normetanephrine levels were 5.80 μg/dl (normal range; 5‐23), 118.65 μg (normal level <350 μg/24 hours), and 228.72 μg (normal level <650 μg/24 hours). CECT abdomen showed well‐defined right adrenal cyst as rounded hypodense lesion measuring approx. 79 × 75 × 78 mm with internal hemorrhage as slight hyperdense echogenic area in it. No fat component or enhancing nodule was identified. Part of adrenal gland was normally visualized. Per abdomen findings were within normal limits.

**FIGURE 3:**
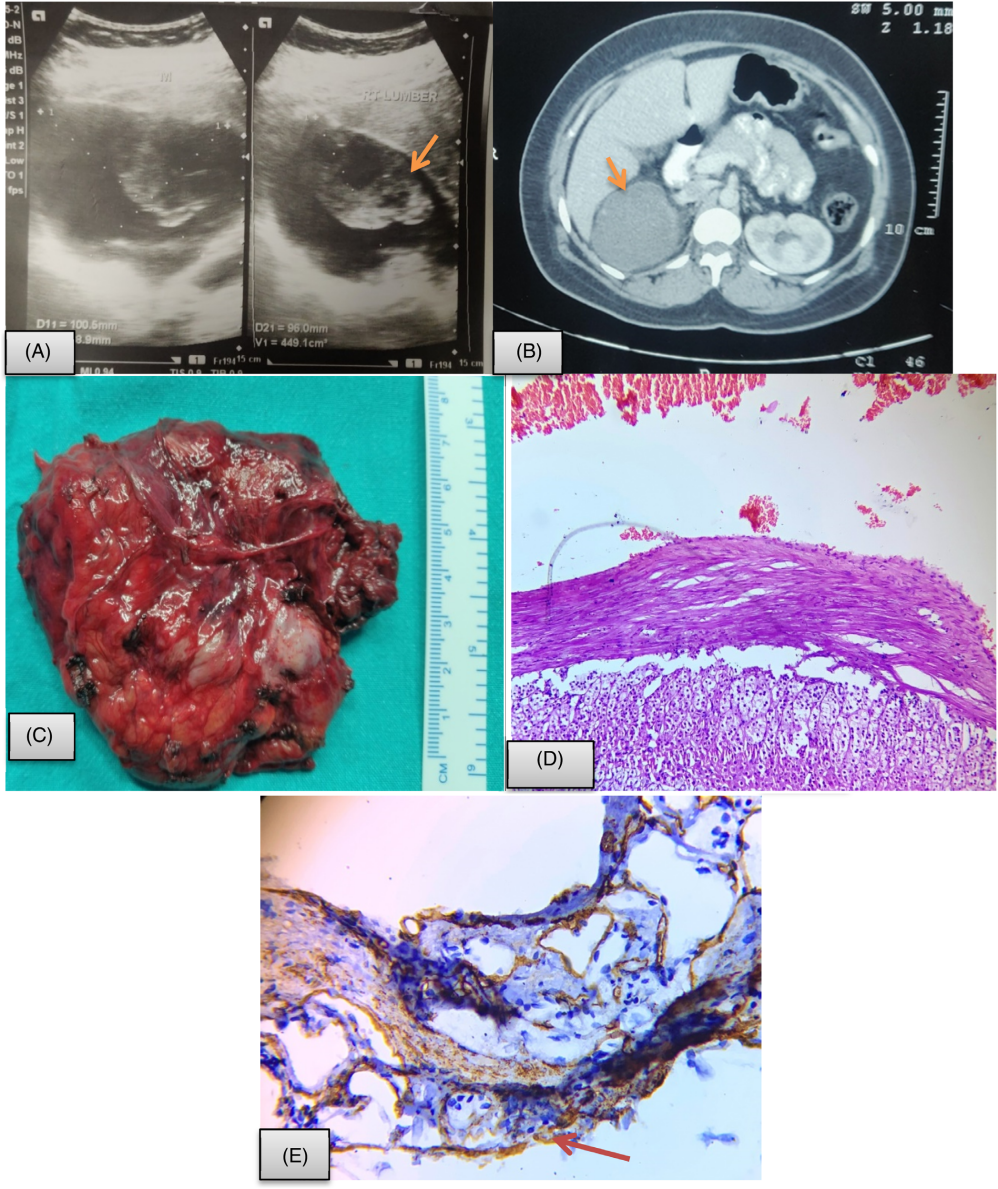
Adrenal endothelial cyst. A, USG abdomen showing a mixed echogenic mass in right suprarenal region (arrow); B, CECT abdomen showed well‐defined right adrenal cyst as rounded hypo dense lesion measuring approx. 79 × 75 × 78 mm with internal haemorrhage as slight hyper dense echogenic area in it (arrow); C, Resected specimen showing rright suprarenal mass measuring 8 × 7 cm with cystic and solid component; D, Histopathological evaluation display cystic structure with fibrocollagenous wall lined by flattened endothelial cells and red blood cells in the lumen with adrenal tissue at the periphery (Hematoxylin and eosin; 40×). E, Immunohistochemistry show CD34 positivity in lining endothelial cells as well as in microvasculature of adrenal tissue (40×)

In view of patient's history of multiple aspiration and guided drain insertion, she was managed with open adrenalectomy via trans‐peritoneal anterior approach. Preoperatively, there was right suprarenal mass measuring 8 × 7 cm with cystic and solid component adhered to surrounding structures. Grossly, specimen weighed 99.6 g, measuring 8 × 6 × 3.5 cm with smooth, congested outer surface. The cut surface showed uniloculated cyst measuring 6 × 4 × 2 cm with areas of hemorrhage. Adrenal tissue could also be identified at places. Microscopic examination revealed endothelial cyst as fibrocollagenous cyst wall lined by flattened endothelial cells along with remnants of adrenocortical tissue (Figure 3).

Case 5 A 40 years female, presented with pain in right hypochondrium and lumbar region for 1 year which was associated with generalized weakness, fatigue, and constipation for the past one month. There was no history of headache, palpitation, diaphoresis, or visual disturbances. On per‐abdominal examination, a lump of size 13 × 10 cm was palpable in right hypochondrium extending to lumbar region, crossing the midline which was of firm to hard in consistency with smooth surface, not palpated separately from liver border. Serum and urinary catecholamines were unremarkable. During investigation, a well‐defined para‐renal 13 × 10 cm septated cyst, of uncertain origin was identified on CT. It was compressing the adjacent bowel and right lobe of liver. The right adrenal gland was not separately identified from the mass, suggesting adrenal origin. The walls of the mass were irregular and thickened at one side showing uniform enhancement. No lymphadenopathy was seen. In view of large size and malignant potential of complex cysts, right open adrenalectomy was performed via anterior transperitoneal approach. Preoperatively, there was a very large predominantly cystic mass measuring 15 × 15 cm with solid areas which was densely adhered to inferior surface of liver.

**FIGURE 4:**
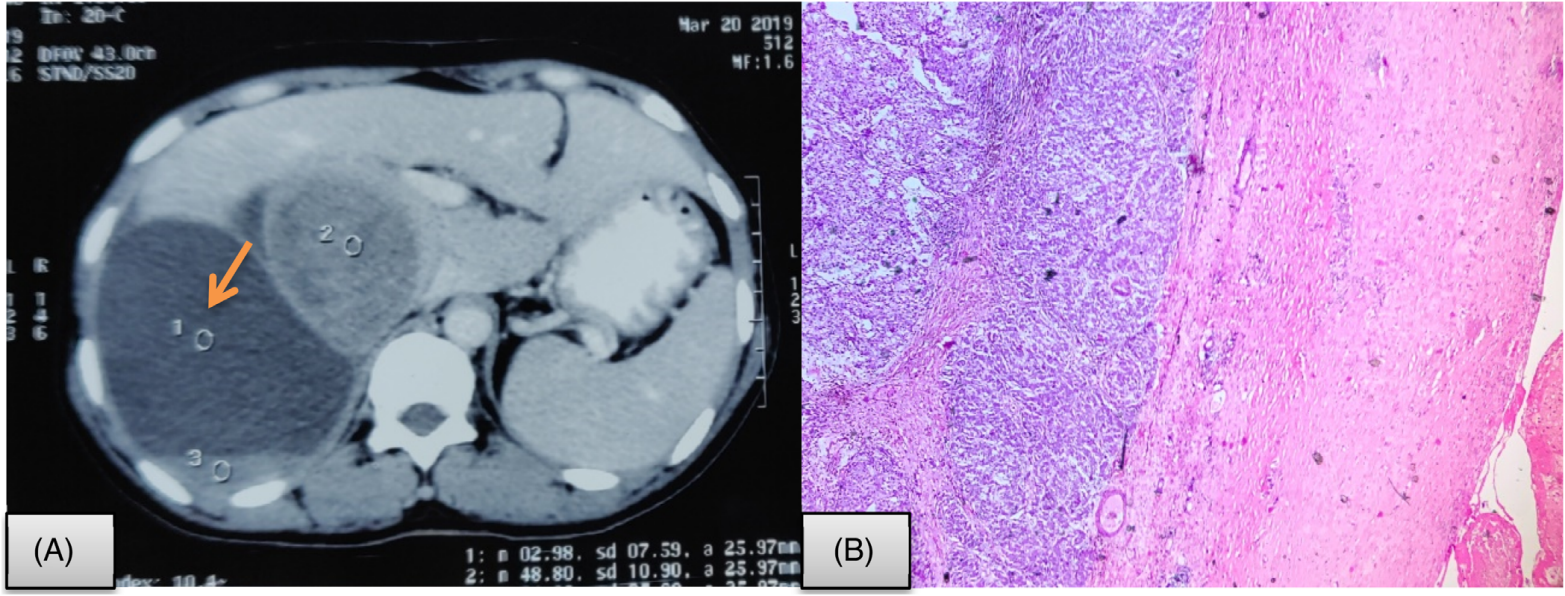
Cystic adrenocortical carcinoma. A, CT scan shows well‐defined para‐renal 13 × 10 cm septated cyst (arrow) compressing the adjacent bowel and right lobe of liver. The right adrenal gland was not separately identified from the mass, suggesting adrenal origin. B, Microscopy shows fibrocollagenous cyst wall with sheets of atypical epithelial cells having high nucleo‐cytoplasmic ratio, pleomorphic nuclei, and moderate amount of eosinophilic cytoplasm (Hematoxylin and eosin; 400×)

Histopathological evaluation revealed a cystic lesion with fibrocollagenous cyst wall and absence of any lining. These were a solid component composed of sheets of atypical cells with resemblance of cortical cells (Figure 4). Mitosis was >5/10 high power field. Areas of necrosis and hemorrhage were also seen along with presence of atypical mitotic figures. The modified Weiss score was 4 suggesting a malignancy. These tumor cells were immunohistochemically positive for synaptophysin, vimentin, melan A, and inhibin with no expression of chromogranin and CK7, CK20. Ki67 proliferation index was 5%. Hence, the case was finally diagnosed as adrenocortical carcinoma arising from a pseudocyst with poor prognostic markers. The patient is lost to follow up.

## 3 DISCUSSION

Adrenal lesions remain uncommon, with incidence in autopsy studies ranging from 0.064 to 0.18%.^5^, ^6^, ^7^ This incidence seems to be growing during the last few decades due to improved and wider use of imaging techniques. These rare lesions are usually benign, nonfunctional, and unilateral and mostly occur in third and sixth decade of life.^3^, ^5^ The reported female to male ratio in literature is 3:1.^8^ They are usually asymptomatic and are discovered incidentally. Approximately 39% of the cases may present with large mass lesion and pain due to hemorrhage or cyst rupture. Rarely (9% of cases) adrenal cysts may be associated with hypertension, probably due to compression of adrenal artery or renal medulla.^9^ In our series, there was a female predominance (male:female 1:3). All four patients were nonfunctional but presented with abdominal pain or fullness with history of repeated aspiration in one of them. This pain is due to stretching of the capsule of adrenal gland.

Terrier and Lecene in 1906 first classified adrenal cysts into hemorrhagic, endothelial, congenital retention, cystic adenomas, and parasitic types.^10^ Many other classifications were formulated eventually. In 1966, Foster classified adrenal cyst into four types based on histological types and incidence: Endothelial cyst (45%), pseudocyst (39%), epithelial cyst (9%), and parasitic cyst (7%). This remains the most accepted classification till date.^11^The origin of formation is still not clear. Endothelial cysts are postulated to be formed by dilated and thrombosed vessels with organization, while pseudocysts are thought to arise from endothelial cysts that undergo repeated hemorrhage and fibrosis.^12^ Endothelial cyst is also known as simple cysts. They are the most common among adrenal cysts in autopsy series with incidence of 45%, but account for only 2% to 24% of clinically symptomatic lesions.^13^ They can be divided as angiomatous and lymphangiomatous. Some theories have also described their origin from pre‐existing vascular hamartoma, hence can be seen in young age also.^20^ A recent study done by Koperski et al^21^ mentioned cystic lymphangiomatous malformations in three broad categories as multicystic, unicystic, and lymphangiomatous cysts with papillary endothelial proliferation. Pseudocysts are most common among all adrenal cysts across different studies with an incidence of 39%.^6^ Adrenal pseudocysts most commonly arise from hemorrhage within the adrenal gland, secondary to extreme stress, birth, trauma, surgery, or malignancy. They have long been known to be associated with malignancy, with an estimated incidence of approximately 7%. Among the malignancies found in adrenal pseudo cysts, adrenocortical carcinoma (ACC) is by far the most common.^14^

As per guidelines, assessment of hormonal levels is to be done in all patients with adrenal incidentaloma. In all the five cases discussed here, hormonal assessment was done and the entire adrenal lesion was nonfunctional. Ultrasonography, CT, and MRI studies have been very effective in recognizing cystic lesions; however, radiologic findings are usually inadequate for the definitive subtyping of a cystic adrenal lesion^15^ or to distinguish benign from malignant entities.^5^ When suspicious, further investigations, that is, biopsies or surgery, are usually performed to rule out malignancy. On imaging studies, the differential diagnoses of cystic adrenal lesions include cysts of liver, spleen, and pancreas, empyema of the gallbladder, and abdominal aneurysms.^16^

Chien et al, in 2008 reviewed the importance of surgical management in patients with adrenal cyst wherein he reported 25 cases of adrenal cyst (16 pseudocysts, 8 endothelial, and 1 epithelial cyst) where seven adrenal pseudocysts were associated with tumor including two pheochromocytomas, one neuroblastoma, one adrenal cortical carcinoma, one adrenal cortical adenoma, one myelolipoma, and one schwannoma. He concluded that because of their heterogeneous etiology and overlapping clinical findings, definite diagnosis relies on extensive sampling and thorough microscopic examination in order to exclude the possibility for coexisting tumor.^17^

Optimum management of adrenal cysts still remain controversy, owing to its low incidence. Surgical management, open or minimally invasive, depends on a surgeon's preference, tumor characteristics, and expertise. Surgery is usually indicated in functional cysts, malignant or potentially malignant cysts, symptomatic cysts of any size, asymptomatic cysts of size more than 5 cm, and those patients with uncertain follow up^13^, ^18^ In our third patient, surgical removal was done in spite of lesion being <4 cm in greatest dimension, due to presence of chronic pain. The remaining cases also underwent adrenalectomy because of larger size as well as presence of symptoms. For lesions measuring more than 8 cm, open adrenalectomy was a better operative approach, as these lesions were found to have dense adhesions with adjacent structures (seen in three out of five patients). The reason for this is not clear, but it may be due to cystic fluid permeating through the capsule causing adhesions with adjacent tissues. Also, it is important to keep in mind that if we plan laparoscopic surgery then we should be prepared for difficult dissection and be ready to covert to open surgery whenever needed. If patient has history of any aspiration or pigtail insertions then that also makes the planes of dissection difficult.

Conservative management is apt in those with uncomplicated/asymptomatic cysts <5 cm.^17^ Though postoperative period of such patients are uneventful and they recover well, a minimum of 18 months of follow up with repeat CT every 6 months is indicated. Aspiration of cyst can be considered as an alternative to surgery in case of surgically unfit patients.^8^, ^13^, ^19^ Marsupialization or decortication have also been tried as alternatives to surgery for large cyst specially those cysts which are adherent to multiple organs where excision may be difficult. Sclerotherapy using absolute alcohol has also been described but it is associated with high recurrence of 30% to 50%.^17^, ^19^

The limitation of our study may seem to be lesser number of cases, however looking at the short study duration (~4 years) as opposed to 20 to 25 years data published in larger studies, the spectrum of adrenal cyst documented in our study seems to be appropriate.

## 4 CONCLUSION

In conclusion, cystic adrenal lesions are not as uncommon as mentioned in past literature. These lesions present with varied manifestations and may also sometime present as diagnostic dilemma. Preoperative evaluation plays an important role to rule out other differential diagnosis. Proper investigation including CT or MRI is essential for defining adrenal cystic lesion and differentiating from cystic lesion of adjacent organs. Benign cyst may harbor underlying malignancy mandating a histopathological evaluation of all cystic adrenal lesions. Surgery is the treatment of choice in symptomatic cases as well asymptomatic cases with a large diameter or increasing dimensions during follow‐up or with any anomaly of adrenal hormones.

## ETHICAL STATEMENT

The present study has been approved by the ethical committee of the institute and consent has been taken from the patients for publication of cases.

## CONFLICT OF INTEREST

There are no conflicts of interest.

## AUTHOR CONTRIBUTIONS

**Divya Goel:** Conceptualization; data curation; formal analysis; methodology. **Loreno Enny:** Conceptualization; data curation; formal analysis; methodology. **chanchal rana:** Conceptualization; data curation; formal analysis; investigation; methodology; project administration; supervision; validation; writing‐original draft; writing‐review and editing. **Pooja Ramakant:** Conceptualization; investigation; writing‐review and editing. **Kulranjan Singh:** Supervision; writing‐review and editing. **Suresh Babu:** Writing‐review and editing. **Anand Mishra:** Supervision; writing‐review and editing.

## Data Availability

The data has not been submitted but will be available on request.
